# Reinforcement learning-enhanced multi-objective optimization for sustainable coal blending in thermal power plants

**DOI:** 10.1371/journal.pone.0331208

**Published:** 2025-09-05

**Authors:** Zhongfeng Li, Lei Liu, Zhenlong Zhao, Shujie Mu, Dong Li, Yuting Zhuo

**Affiliations:** 1 School of Electrical Engineering, Yingkou Institute of Technology, Yingkou, Liaoning, People’s Republic of China; 2 School of Electronic and Information Engineering, University of Science and Technology Liaoning, Anshan, Liaoning, People’s Republic of China; 3 Huaneng Yingkou Xianrendao Thermal Power Co., Yingkou, Liaoning, People’s Republic of China; 4 School of Chemical Engineering, University of New South Wales, Sydney, New South Wales, Australia; Indian Institute of Technology Kanpur, INDIA

## Abstract

Coal blending in thermal power plants is a complex multi-objective challenge involving economic, operational and environmental considerations. This study presents a Q-learning-enhanced NSGA-II (QLNSGA-II) algorithm that integrates the adaptive policy optimization of Q-learning with the elitist selection of NSGA-II to dynamically adjust crossover and mutation rates based on real-time performance metrics. A physics-based objective function takes into account the thermodynamics of ash fusion and the kinetics of pollutant emission, ensuring compliance with combustion efficiency and NO_*x*_ limits. Benchmark tests on the Walking Fish Group (WFG) and Unconstrained Function (UF) suites show that QLNSGA-II achieves a 12.7% improvement in Inverted Generational Distance (IGD) and a 9.3% improvement in Hypervolume (HV) compared to prevailing algorithms. Industrial validation at the Huaneng Yingkou power plant confirms a 14.7% reduction in fuel cost and a 41% reduction in slagging incidence over conventional blending methods, backed by 12 months of operational data. Other benefits include a 24.8% reduction in sulphur content, a 6.9% increase in the plant’s net heat rate and annual savings of RMB 12.3 million, 2,150 tonnes of limestone and 38,500 tonnes of CO_2_-equivalent emissions. These results highlight QLNSGA-II as a scalable, robust solution for multi-objective coal blending, offering a promising way to improve the efficiency and sustainability of coal-fired power generation.

## 1 Introduction

Thermal power generation remains a cornerstone of global energy systems, accounting for approximately 36% of worldwide electricity production[1,2], with coal-fired plants contributing over 70% of this share in coal-dependent economies such as China and India [3–5]. The practice of coal blending emerged in the late 1970s as a strategic response to declining coal quality and supply volatility, initially focusing on empirical mixtures to stabilize boiler operations [6–8]. Over the decades, this practice has evolved into a sophisticated optimization challenge, driven by the need to balance conflicting requirements: minimizing fuel costs, adhering to stringent emission regulations (e.g., China’s Ultra-Low Emission standards), and maintaining combustion stability across diverse coal properties[9,10]. Modern blending frameworks now incorporate computational models to address these multidimensional constraints, marking a shift from heuristic-based approaches to data-driven decision making [11–13].

Contemporary research in coal blending optimization predominantly focuses on algorithmic advancements to manage its inherent complexity—a high-dimensional problem space with 5–7 interdependent variables (e.g., calorific value, ash fusion characteristics) and nonlinear objective relationships [14]. As summarized in Table 1, existing methodologies span from traditional mathematical programming to hybrid artificial intelligence techniques. Linear programming models, while computationally efficient, oversimplify combustion dynamics and ash interaction effects [15]. Evolutionary algorithms like NSGA-II excel in multi-objective optimization but are limited in adaptability to real-time operational shifts [16]. Particle swarm optimization (PSO) variants demonstrate rapid convergence yet often stagnate in local optima when handling more than four objectives [17]. Recent hybrid approaches combining neural networks with metaheuristics [18] show promise in predictive modeling, but require extensive training data and lack interpretability for plant operators. A critical challenge remains in dynamically balancing exploration and exploitation during optimization, particularly in fluctuating coal markets and regulatory environments [2,19].

**Table 1:** Summary of optimization algorithms for coal blending in thermal power plants.

Algorithm Type	Disturbances	Constraints	Objectives	Solving Method	Ref.
Traditional Mathematical Programming	Variability in coal quality	Cost, emission limits	Cost minimization, emission control	Linear/Non-linear programming	[15,20]
Genetic Algorithms (GA)	Variable coal blend ratios	Quality, cost constraints	Optimize cost, quality	NSGA-II, GA with constraint handling	[21–23]
Particle Swarm Optimization (PSO)	Uncertain supply conditions	Emission, quality	Cost, emission reduction	MOPSO, Hybrid PSO	[2,17]
Multi-Objective Evolutionary Algorithms (MOEAs)	Coal property fluctuations	Economic, environmental objectives	Balance cost, emission goals	MOEA/D, NSGA-II	[6,24]
Case-Based Reasoning (CBR)	Real-time combustion changes	Quality, emission limits	Emission control, operational stability	Online case-based reasoning	[16]
Hybrid Optimization Approaches	Environmental regulations	Cost, quality constraints	Cost and emission reduction	Hybrid models combining PSO, GA, ANN	[18,25]
Artificial Neural Networks (ANN)	Dynamic quality adjustments	Quality, emissions	Adaptive blend for emissions, quality	ANN and experimental design	[7,26]

Over the past decade, the field of multi-objective optimization algorithms (MOOAs) has progressed rapidly [27–29], driven by the practical necessity to solve increasingly high-dimensional and complex engineering problems [30,31]. A diverse array of recent advances—including adaptive predator–prey frameworks, weighted average mechanisms, and archive-boosted strategies—has markedly improved the convergence and diversity of Pareto solutions across benchmark and real-world scenarios [32–34]. Furthermore, novel algorithmic designs such as the Multi-objective Runge–Kutta Optimizer (MORKO) [35], Many-Objective Multi-Verse Optimizer (MaOMVO) [36], and nature-inspired variants like MaODA and MaOGOA have significantly enhanced scalability and robustness, particularly for problems characterized by numerous conflicting objectives and practical engineering constraints [37,38].

Despite these substantial methodological gains, current literature continues to grapple with several unresolved issues that directly affect application to domains such as coal blending optimization. Notably, the challenge of balancing solution diversity with convergence efficiency in high-dimensional search spaces remains at the forefront of research. Additionally, the integration of real-time, dynamic operational data into optimization models is still an open problem, as most algorithms assume static environments that may not reflect industrial realities . Another persistent limitation lies in the generalizability of these frameworks across heterogeneous system configurations and varying material properties. Although reliability-driven approaches and swarm intelligence-based methods continue to evolve, their adoption for domain-specific objectives—such as the simultaneous minimization of fuel cost and pollutant emissions in power generation—has yet to be fully realized.

Coal blending optimization holds both academic and industrial significance due to its broad impact on economic viability, environmental compliance, and operational reliability [9,39]. Despite steady methodological progress, the literature continues to highlight three core challenges: achieving a balance between solution diversity and convergence speed in high-dimensional objective spaces [40]; integrating real-time market and operational data into predominantly static optimization models; and generalizing algorithmic frameworks to accommodate heterogeneous boiler configurations and varying coal properties. Against this backdrop, reinforcement learning (RL) has emerged as a promising approach for introducing adaptive, data-driven decision-making into multi-objective optimization, particularly by overcoming the rigidity of static models. However, its practical application in coal blending, especially in synergy with population-based evolutionary algorithms to enable real-time policy updates and dynamic constraint handling, remains largely unexplored [41,42]. Bridging the gap between these algorithmic advances and domain-specific requirements is thus essential for addressing the complex, evolving optimization demands of modern power generation systems.

This study addresses these gaps through three principal contributions:

1. **Adaptive Multi-Objective Framework:** The proposed QLNSGA-II algorithm synergizes Q-learning’s policy optimization with NSGA-II’s elitist selection, enabling dynamic adjustment of crossover/mutation probabilities based on real-time solution quality metrics.
2. **High-Dimensional Constraint Handling:** A physics-informed objective function incorporates ash fusion thermodynamics and pollutant emission kinetics, resolving conflicts between combustion efficiency (η>92%) and NO_*x*_ emissions ($<50\;{\text{mg/Nm}}^{3}$).
3. **Industrial Validation:** Implementation at Huaneng Yingkou Power Plant demonstrates a 14.7% cost reduction and a 41% lower slagging incidence compared to conventional blends, validated through 12-month operational data and emission monitoring.

The subsequent sections detail the mathematical formulation of coal blending optimization (Sect 2), QLNSGA-II’s algorithmic architecture (Sect 3), benchmark validation against WFG/UF test suites (Sect 4), and empirical results from full-scale plant trials (Sect 5). Concluding remarks outline future directions for RL-enhanced optimization in energy systems.

## 2 Coal blending optimization model

The multi-objective optimization model for coal blending addresses three critical aspects: economic viability (*F*_*e*_), operational safety (*F*_*s*_), and environmental compliance (*F*_*p*_). The formulation integrates physicochemical coal properties with industrial constraints through additive blending principles [6,22]:

$$\min 𝐅(𝐱)=[\begin{array}{c} F_{e}(𝐱)\\ F_{s}(𝐱)\\ F_{p}(𝐱) \end{array}]\;\text{s.t.}\;Q_{L}\leq \sum_{i=1}^{n}x_{i}Q_{i}\leq Q_{U},V_{L}\leq \sum_{i=1}^{n}x_{i}V_{i}\leq V_{U},\;M_{L}\leq \sum_{i=1}^{n}x_{i}M_{i}\leq M_{U},ST_{L}\leq \sum_{i=1}^{n}x_{i}ST_{i}\leq ST_{U},\;A_{L}\leq \sum_{i=1}^{n}x_{i}A_{i}\leq A_{U},\sum_{i=1}^{n}x_{i}=1,\;x_{i}\geq 0\;\forall i=1,2,\ldots ,n.$$ (1)

### 2.1 Objective functions

The economic objective *F*_*e*_ minimizes blending costs normalized to market prices:

$$F_{e}(𝐱)=\theta \sum_{i=1}^{n}\frac{x_{i}P_{i}}{P_{m}}$$ (2)

The safety objective *F*_*s*_ combines deviations from target boiler parameters (*Q*_*d*_, $V_{d}$, *M*_*d*_) and ash fusion risks:

$$F_{s}(𝐱)=\alpha (\underset{\text{Calorific stability}}{\underbrace{\sum_{i=1}^{n}\frac{x_{i}Q_{i}-Q_{d}}{Q_{d}}}}+\underset{\text{Volatile consistency}}{\underbrace{\sum_{i=1}^{n}\frac{x_{i}V_{i}-V_{d}}{V_{d}}}}+\underset{\text{Moisture control}}{\underbrace{\sum_{i=1}^{n}\frac{x_{i}M_{i}-M_{d}}{M_{d}}}})\;\;+\beta (\underset{\text{Sulfur limit}}{\underbrace{\sum_{i=1}^{n}\frac{x_{i}S_{i}-S_{\min}}{S_{\max}-S_{\min}}}}-\underset{\text{Slagging risk}}{\underbrace{\sum_{i=1}^{n}\frac{x_{i}ST_{i}-ST_{\min}}{ST_{\max}-ST_{\min}}}})$$ (3)

The environmental objective *F*_*p*_ comprehensively evaluates the impacts from sulfur emissions (*S*_*i*_), ash disposal (*A*_*i*_), as well as key gaseous pollutants including NO_*x*_ and CO_2_-equivalent emissions:

$$F_{p}(𝐱)=\gamma (\underset{{\text{SO}}_{x}\text{ mitigation}}{\underbrace{\sum_{i=1}^{n}\frac{x_{i}S_{i}-S_{\min}}{S_{\max}-S_{\min}}}}+\underset{\text{Ash reduction}}{\underbrace{\sum_{i=1}^{n}\frac{x_{i}A_{i}-A_{\min}}{A_{\max}-A_{\min}}}}+\delta_{1}\underset{{\text{NO}}_{x}\text{ emissions}}{\underbrace{\sum_{i=1}^{n}\frac{x_{i}N_{i}-N_{\min}}{N_{\max}-N_{\min}}}}+\delta_{2}\underset{{\text{CO}}_{2}\text{-eq emissions}}{\underbrace{\sum_{i=1}^{n}\frac{x_{i}C_{i}-C_{\min}}{C_{\max}-C_{\min}}}})$$ (4)

where, *N*_*i*_ and *C*_*i*_ denote the NO_*x*_ and CO_2_-equivalent emission factors for each coal type, with $\delta_{1}$ and $\delta_{2}$ as their respective weights. This approach enables direct evaluation and minimization of atmospheric pollutants alongside traditional ash and SO_*x*_ indices, reflecting the increasing importance of multi-pollutant emission control in modern coal utilization.

### 2.2 Parameter calibration

Weight coefficients (*θ*, *α*, *β*, *γ*) were determined through iterative sensitivity analysis using historical plant data (2018–2023), prioritizing economic factors (θ=0.6) while ensuring safety (α=0.25, β=0.15) and environmental (γ=0.35) constraints. The newly introduced NO_*x*_ and CO_2_-eq weights ($\delta_{1}$, $\delta_{2}$) were calibrated in line with site emission benchmarks and the latest regulatory targets. Boundary conditions ($Q_{L}/Q_{U}$, etc.) align with China’s GB/T 15224.1-2021 coal standards and plant-specific boiler specifications.

### 2.3 Decision variables

The vector $𝐱=[x_{1},\ldots ,x_{n}{]}^{⊤}$ represents coal proportions, constrained by:

- Physicochemical limits: *Q*_*i*_ (calorific value), $V_{i}$ (volatiles), *M*_*i*_ (moisture), *S*_*i*_ (sulfur), *ST*_*i*_ (ash melting point), *A*_*i*_ (ash content) , *N*_*i*_ (NO_*x*_ factor), *C*_*i*_ (CO_2_ factor)
- Operational thresholds: *Q*_*d*_ = 4525 kJ/kg, $V_{d}=25.5\%$, $M_{d}=20.65\%$
- Environmental caps: $S_{\min}=0.14\%$, $S_{\max}=0.72\%$, $A_{\max}=24.54\%$ , $N_{\max}$, $C_{\max}$ as required

The model generates Pareto-optimal blends balancing cost (RMB/ton-MJ), slagging risk (ST deviation), and emission penalties (S/A indices, NO_*x*_, CO_2_-eq), validated through plant trials in Sect 5.

## 3 The proposed QLNSGA-II algorithm

### 3.1 Standard NSGA-II

NSGA-II is a well-established algorithm for multi-objective optimization [43,44], and it has proven effective for coal blending in thermal power generation due to its capability to handle complex, multi-objective problems. The pseudo-code of the NSGA-II algorithm is presented in Algorithm 1, detailing the process from initialization to final population refinement.

**Algorithm 1. Pseudo-code of NSGA-II algorithm.**



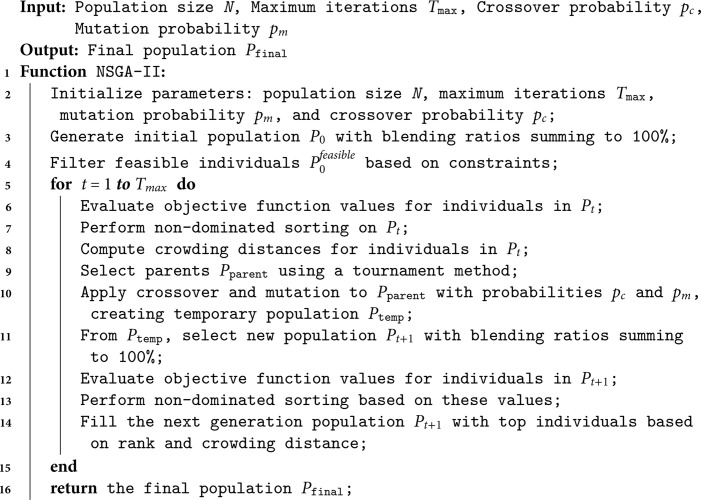



### 3.2 Q-Learning enhanced NSGA-II

The proposed algorithm embeds Q-learning into the operator selection process of NSGA-II, enabling dynamic adaptation of crossover and mutation strategies according to evolving population characteristics [41,45,46]. Unlike traditional NSGA-II, which relies on fixed probabilities, the QNSGA-II approach leverages historical search experience to select operators more intelligently, thus improving both convergence and diversity [47,48].

#### 3.2.1 State-action-reward framework.

The state space is constructed by partitioning the population with respect to the median values of makespan and total energy consumption (TEC), resulting in four states as shown in Table 2. Each state represents a distinct region in the Pareto space and informs the operator selection process via Q-learning.

**Table 2:** Q-Learning state definition based on objective medians.

State	Makespan < Median	Makespan ≥ Median	TEC < Median	TEC ≥ Median
1	✓		✓	
2	✓			✓
3		✓	✓	
4		✓		✓

Six neighborhood operators are defined as actions, summarized in Table 3. Each operator modifies the solution in a specific manner, including both crossover and mutation strategies, ensuring adequate search capability in different regions of the solution space.

**Table 3:** Neighborhood operators (actions) in Q-learning.

Action	Description
1	Two-point crossover (arithmetic)
2	Two-point crossover (blend)
3	Simulated binary crossover (low index)
4	Simulated binary crossover (high index)
5	Polynomial mutation (high probability)
6	Polynomial mutation (low probability)

The reward for each action is calculated by combining the dominance relationship and normalized objective improvement:

$$R_{j}=\omega_{1}\frac{1}{\text{rank}(X_{j})}+\omega_{2}\sum_{k=1}^{K}\frac{f_{\max}^{\;k}-f_{j}^{\;k}}{f_{\max}^{\;k}-f_{\min}^{\;k}},$$ (5)

where $\omega_{1}+\omega_{2}=1$ controls the balance between convergence and diversity incentives.

Table 4 illustrates the evolution of Q-values for each state-action pair across generations, while Table 5 provides statistics for operator selection frequencies. These results confirm that the Q-learning mechanism gradually biases operator selection toward actions that yield higher long-term rewards.

**Table 4:** Q-Table values for each state-action pair at selected generations (mean over 20 runs).

State	1	2	3	4	5	6
**Generation 1**						
1	0.007	0.012	0.009	0.006	0.011	0.008
2	0.008	0.009	0.010	0.010	0.008	0.009
3	0.010	0.007	0.011	0.008	0.009	0.010
4	0.009	0.008	0.010	0.011	0.007	0.009
**Generation 50**						
1	0.131	0.112	0.146	0.123	0.110	0.140
2	0.117	0.108	0.133	0.121	0.118	0.123
3	0.129	0.141	0.119	0.124	0.125	0.129
4	0.114	0.119	0.122	0.138	0.120	0.128
**Generation 100**						
1	0.278	0.247	0.298	0.260	0.225	0.289
2	0.243	0.229	0.265	0.259	0.241	0.258
3	0.270	0.284	0.238	0.263	0.249	0.272
4	0.233	0.241	0.256	0.282	0.237	0.255

**Table 5:** Selection frequency (%) of each action (average over 20 runs).

Action	1	2	3	4	5	6
Frequency (%)	17.8	14.9	15.7	20.3	16.2	15.1

Parameter sensitivity is further analyzed using a Taguchi L9 orthogonal array (Table 6). The Q-learning parameters, particularly the learning rate and discount factor, are shown to substantially influence convergence and diversity, with best performance at *P*_*c*_ = 0.8, *P*_*m*_ = 0.1, α=0.1, γ=0.9.

**Table 6:** Taguchi L9 orthogonal array for parameter sensitivity.

Group	*P*_*c*_	*P*_*m*_	*α*	*γ*
1	0.7	0.05	0.1	0.7
2	0.7	0.10	0.2	0.8
3	0.7	0.15	0.3	0.9
4	0.8	0.05	0.2	0.9
5	0.8	0.10	0.3	0.7
6	0.8	0.15	0.1	0.8
7	0.9	0.05	0.3	0.8
8	0.9	0.10	0.1	0.9
9	0.9	0.15	0.2	0.7

#### 3.2.2 Dynamic operator selection.

At each generation, operator selection is adaptively guided by the Q-learning mechanism. Q-values are updated according to

$$Q(s_{t},a_{t})\leftarrow Q(s_{t},a_{t})+\delta \left[R_{t+1}+\eta \max_{a_{t+1}}Q(s_{t+1},a_{t+1})-Q(s_{t},a_{t})\right],$$ (6)

where *δ* and *η* represent the learning rate and discount factor. The dynamic *ε*-greedy strategy

$$\epsilon (g)=\frac{1}{1+\exp\left(1.2-\frac{5g}{G_{\max}}\right)}$$ (7)

ensures adequate exploration in early generations and more exploitation as the search progresses.

Algorithm 2 outlines the iterative process, in which each individual is assigned a state, selects an operator via *ε*-greedy, and updates the Q-table based on observed rewards. This adaptive mechanism gradually guides the search towards more effective operator combinations.

**Algorithm 2. Q-Learning enhanced NSGA-II with adaptive operator selection.**



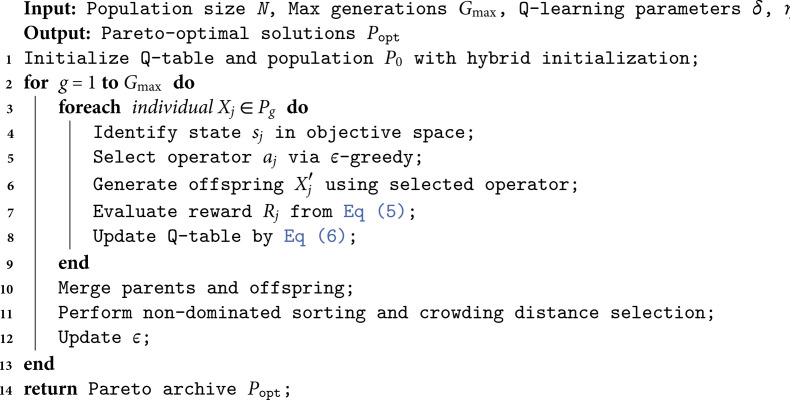



#### 3.2.3 Adaptive constraint handling.

Constraint handling is seamlessly integrated into the Q-learning reward system. When an operator generates a feasible solution, it receives an additional reward,

$$R_{\text{feas}}=1-\frac{\sum_{c=1}^{C}\max(0,g_{c}(X))}{\sum_{c=1}^{C}g_{c}^{\max}},$$ (8)

where *g*_*c*_(*X*) and $g_{c}^{\max}$ denote the violation and maximum violation of constraint *c*. This approach biases operator selection toward feasible regions, obviating the need for penalty parameter tuning and naturally balancing objectives with constraint satisfaction.

The initial population is constructed using a hybrid strategy: 60% of individuals are generated via Tent chaotic mapping to promote diversity, while the remaining 40% are constructed with domain heuristics to improve solution quality. This approach accelerates convergence and ensures a broad search space from the outset.

## 4 Evaluation of the proposed QLNSGA-II

The performance of the proposed QLNSGA-II was systematically evaluated by benchmarking it against five established multi-objective optimization algorithms: MOPSO [49], rNSGA-II [50], IMMOEAD [51], MOEAD-FRR-MAB [52], and dMOPSO [53]. The selection of these algorithms was made to ensure a fair and comprehensive comparison with recent and widely recognized baselines in the field.

MOPSO and dMOPSO are two widely-cited variants of multi-objective particle swarm optimization, with dMOPSO representing an advanced co-evolutionary framework and MOPSO serving as a classical PSO-based baseline. rNSGA-II is a reference-point-based variant of NSGA-II and remains influential due to its distinctive selection and sorting mechanisms. IMMOEAD and MOEAD-FRR-MAB are both state-of-the-art decomposition-based evolutionary algorithms that incorporate adaptive strategies for operator selection and constraint handling, representing the latest advances in MOEA/D research. The inclusion of these algorithms provides coverage of both population-based and decomposition-based paradigms, as well as representative works from the most recent literature.

To evaluate the effectiveness of QLNSGA-II in terms of convergence and diversity, experiments were carried out using the WFG and UF benchmark test suites [54], which are standard platforms for assessing multi-objective optimization algorithms. Both suites are known for presenting complex challenges—such as multi-modality, non-separability, and scalability in the number of objectives—thereby enabling a thorough assessment of the algorithm’s generalization capabilities.

Uniform parameter settings were maintained across all comparative methods, as summarized in Table 7, to ensure a fair and unbiased evaluation environment. For all algorithms, the population size was set to 100, the maximum number of generations was 10,000, and the crossover and mutation probabilities were fixed at 0.9 and 1/*D*, respectively, where *D* denotes the number of decision variables. Reference and ideal points were selected in accordance with the settings recommended in the most recent comparative studies, thereby allowing for direct comparison of performance based on Hypervolume (HV) and Inverted Generational Distance (IGD) metrics.

**Table 7:** Parameter settings and performance evaluation criteria for WFG and UF test functions.

Test Function	*M*	*D*	Population Size	Max Generations	Crossover Probability	Mutation Probability	Reference Point (HV)	Ideal Point (IGD)
WFG1	3	12	100	10000	0.9	1/*D*	(2, 4, 6)	(0, 0, 0)
WFG2	3	12	100	10000	0.9	1/*D*	(2, 4, 6)	(0, 0, 0)
WFG3	3	12	100	10000	0.9	1/*D*	(2, 4, 6)	(0, 0, 0)
WFG4	3	12	100	10000	0.9	1/*D*	(2, 4, 6)	(0, 0, 0)
WFG5	3	12	100	10000	0.9	1/*D*	(2, 4, 6)	(0, 0, 0)
WFG6	3	12	100	10000	0.9	1/*D*	(2, 4, 6)	(0, 0, 0)
WFG7	3	12	100	10000	0.9	1/*D*	(2, 4, 6)	(0, 0, 0)
WFG8	3	12	100	10000	0.9	1/*D*	(2, 4, 6)	(0, 0, 0)
WFG9	3	12	100	10000	0.9	1/*D*	(2, 4, 6)	(0, 0, 0)
UF1	2	30	100	10000	0.9	1/*D*	(1, 1)	(0, 0)
UF2	2	30	100	10000	0.9	1/*D*	(1, 1)	(0, 0)
UF3	2	30	100	10000	0.9	1/*D*	(1, 1)	(0, 0)
UF4	2	30	100	10000	0.9	1/*D*	(1, 1)	(0, 0)
UF5	2	30	100	10000	0.9	1/*D*	(1, 1)	(0, 0)
UF6	2	30	100	10000	0.9	1/*D*	(1, 1)	(0, 0)
UF7	2	30	100	10000	0.9	1/*D*	(1, 1)	(0, 0)
UF8	3	30	100	10000	0.9	1/*D*	(2, 4, 6)	(0, 0, 0)
UF9	3	30	100	10000	0.9	1/*D*	(2, 4, 6)	(0, 0, 0)
UF10	3	30	100	10000	0.9	1/*D*	(2, 4, 6)	(0, 0, 0)

All experiments were performed on a standard personal desktop computer equipped with an Intel Core i7 processor, 32 GB RAM, and a 512 GB SSD, running Windows 10. The algorithms were implemented and executed in MATLAB R2022b.

### 4.1 Results and discussion on IGD and HV performance

The proposed QLNSGA-II algorithm was rigorously evaluated on the PlatEMO platform against five state-of-the-art multi-objective optimization algorithms using the WFG and UF benchmark suites [55,56]. Statistical results from 30 independent runs, presented in Tables 8 and 9, demonstrate QLNSGA-II’s superior convergence and diversity characteristics through the IGD and HV metrics.

**Table 8:** IGD performance comparison of QLNSGAII against MOPSO, rNSGAII, IMMOEAD, MOEADFRRMAB, and dMOPSO on WFG and UF test functions.

Problem	M	D	MOPSO	rNSGAII	IMMOEAD	MOEADFRRMAB	dMOPSO	QLNSGAII
WFG1	3	12	1.7770e+0 (7.99e-2) -	8.9060e-1 (2.13e-1) -	1.4262e+0 (9.42e-2) -	1.5928e+0 (4.17e-2) -	1.5353e+0 (1.83e-2) -	**7.1033e-1 (7.47e-2)**
WFG2	3	12	**2.2104e-1 (1.10e-2)** +	1.0580e+0 (3.19e-2) -	3.3489e-1 (1.08e-2) -	3.7918e-1 (1.95e-2) -	4.0509e-1 (2.42e-2) -	2.4021e-1 (1.17e-2)
WFG3	3	12	3.0797e-1 (7.96e-2) -	1.4170e+0 (6.38e-2) -	2.5538e-1 (1.61e-2) -	3.0874e-1 (3.96e-2) -	3.8235e-1 (8.00e-2) -	**1.4381e-1 (1.75e-2)**
WFG4	3	12	4.0278e-1 (3.72e-2) -	1.6258e+0 (3.27e-1) -	3.6270e-1 (5.09e-3) -	4.0675e-1 (1.89e-2) -	3.9577e-1 (2.22e-2) -	**2.9761e-1 (9.49e-3)**
WFG5	3	12	6.5832e-1 (5.59e-2) -	2.0789e+0 (3.88e-1) -	3.5858e-1 (1.11e-2) -	3.3645e-1 (4.11e-3) -	3.9178e-1 (2.55e-2) -	**3.0304e-1 (1.25e-2)**
WFG6	3	12	3.4481e-1 (4.26e-2) =	1.8975e+0 (2.18e-1) -	3.8872e-1 (1.19e-2) -	4.1760e-1 (3.02e-2) -	4.3570e-1 (3.81e-2) -	**3.4468e-1 (1.90e-2)**
WFG7	3	12	3.7612e-1 (4.14e-2) -	1.9028e+0 (1.44e-1) -	3.6843e-1 (6.85e-3) -	4.0269e-1 (2.17e-2) -	4.2826e-1 (1.49e-2) -	**2.9438e-1 (1.18e-2)**
WFG8	3	12	5.9532e-1 (3.78e-2) -	1.8624e+0 (1.59e-1) -	4.2818e-1 (8.81e-3) -	4.7468e-1 (2.13e-2) -	6.1803e-1 (3.98e-2) -	**3.9123e-1 (1.13e-2)**
WFG9	3	12	3.2455e-1 (3.12e-2) -	1.5428e+0 (3.59e-1) -	3.4540e-1 (7.48e-3) -	3.8717e-1 (3.54e-2) -	3.4739e-1 (2.06e-2) -	**2.9349e-1 (1.20e-2)**
UF1	2	30	3.0460e-1 (6.79e-2) -	3.1769e-1 (6.42e-2) -	2.0467e-1 (8.26e-2) -	1.8500e-1 (4.46e-2) -	6.6512e-1 (1.09e-1) -	**1.0921e-1 (2.11e-2)**
UF2	2	30	1.0560e-1 (1.87e-2) -	3.1526e-1 (2.22e-2) -	1.0652e-1 (3.79e-2) -	8.2458e-2 (9.79e-3) -	1.0053e-1 (8.73e-3) -	**6.4128e-2 (3.92e-3)**
UF3	2	30	3.2877e-1 (2.48e-2) +	3.5782e-1 (4.68e-2) +	**3.0785e-1 (3.20e-2)** +	3.3631e-1 (5.04e-2) +	3.3969e-1 (7.44e-3) +	4.2967e-1 (2.42e-2)
UF4	2	30	1.5966e-1 (1.24e-2) -	3.7911e-1 (1.44e-2) -	1.0114e-1 (4.08e-3) -	8.5948e-2 (7.60e-3) -	1.3312e-1 (5.67e-3) -	**7.7462e-2 (3.28e-3)**
UF5	2	30	1.7753e+0 (4.46e-1) -	**6.9104e-1 (2.36e-1)** =	1.4515e+0 (2.51e-1) -	1.8579e+0 (3.84e-1) -	2.9194e+0 (2.93e-1) -	6.9491e-1 (1.73e-1)
UF6	2	30	1.0088e+0 (3.38e-1) -	**4.0261e-1 (1.07e-1)** +	6.6350e-1 (2.12e-1) -	8.2179e-1 (1.49e-1) -	1.9865e+0 (6.38e-1) -	4.8834e-1 (8.24e-2)
UF7	2	30	3.9495e-1 (1.19e-1) -	3.2734e-1 (1.14e-1) -	3.8459e-1 (1.78e-1) -	1.8661e-1 (8.37e-2) -	4.2409e-1 (1.25e-1) -	**1.6470e-1 (1.13e-1)**
UF8	3	30	3.1432e-1 (6.37e-2) =	5.0381e-1 (6.96e-2) -	**2.6993e-1 (4.78e-3)** +	3.7958e-1 (4.96e-2) -	3.7034e-1 (4.21e-2) -	3.1154e-1 (4.37e-2)
UF9	3	30	5.0422e-1 (4.83e-2) -	4.5666e-1 (5.25e-2) -	4.6799e-1 (7.32e-2) -	4.9393e-1 (6.35e-2) -	6.4304e-1 (4.89e-2) -	**4.1838e-1 (5.45e-2)**
UF10	3	30	8.7027e-1 (1.34e-1) +	**7.4443e-1 (2.38e-1)** +	1.1150e+0 (2.55e-1) =	3.2518e+0 (3.66e-1) -	9.2465e-1 (1.17e-1) =	1.0433e+0 (2.90e-1)
+/-/=			3/14/2	3/15/1	2/16/1	1/18/0	1/17/1	

**Table 9:** HV performance comparison of QLNSGAII against MOPSO, rNSGAII, IMMOEAD, MOEADFRRMAB, and dMOPSO on WFG and UF test functions.

Problem	M	D	MOPSO	rNSGAII	IMMOEAD	MOEADFRRMAB	dMOPSO	QLNSGAII
WFG1	3	12	1.6921e-1 (2.67e-2) -	5.1416e-1 (4.74e-2) -	2.2296e-1 (4.29e-2) -	2.4388e-1 (1.98e-2) -	2.8349e-1 (6.36e-3) -	**6.2017e-1 (3.25e-2)**
WFG2	3	12	8.8240e-1 (9.31e-3) -	5.5665e-1 (4.30e-3) -	8.5122e-1 (8.65e-3) -	8.6206e-1 (1.28e-2) -	7.7077e-1 (1.39e-2) -	**8.9993e-1 (5.37e-3)**
WFG3	3	12	2.6717e-1 (3.01e-2) -	1.6379e-1 (9.60e-3) -	3.0077e-1 (9.02e-3) -	2.7336e-1 (1.88e-2) -	2.5841e-1 (1.31e-2) -	**3.6965e-1 (7.64e-3)**
WFG4	3	12	4.2646e-1 (1.42e-2) -	2.0647e-1 (2.42e-2) -	4.7526e-1 (5.12e-3) -	4.4633e-1 (6.60e-3) -	4.2240e-1 (8.55e-3) -	**4.8978e-1 (5.09e-3)**
WFG5	3	12	2.7316e-1 (1.53e-2) -	1.6870e-1 (2.14e-2) -	4.4418e-1 (9.33e-3) -	4.6142e-1 (3.38e-3) -	4.1053e-1 (1.67e-2) -	**4.6909e-1 (5.83e-3)**
WFG6	3	12	**4.5101e-1 (1.92e-2)** =	1.4711e-1 (1.86e-2) -	4.4235e-1 (1.27e-2) =	4.1629e-1 (4.29e-2) =	3.7959e-1 (2.41e-2) -	4.4216e-1 (1.53e-2)
WFG7	3	12	4.2939e-1 (1.45e-2) -	1.5797e-1 (2.03e-2) -	4.7532e-1 (5.95e-3) -	4.5847e-1 (1.69e-2) -	3.6993e-1 (8.78e-3) -	**5.0015e-1 (6.07e-3)**
WFG8	3	12	3.1745e-1 (1.20e-2) -	1.3484e-1 (1.76e-2) -	3.9558e-1 (4.28e-3) -	3.5358e-1 (1.59e-2) -	2.8773e-1 (1.13e-2) -	**4.1590e-1 (6.21e-3)**
WFG9	3	12	4.5697e-1 (1.19e-2) -	2.2380e-1 (3.29e-2) -	4.7015e-1 (6.52e-3) -	4.2341e-1 (4.67e-2) -	4.3316e-1 (1.42e-2) -	**4.8066e-1 (8.06e-3)**
UF1	2	30	3.2612e-1 (7.09e-2) -	4.2981e-1 (4.32e-2) -	4.7356e-1 (4.48e-2) -	4.6221e-1 (5.99e-2) -	6.3275e-2 (6.03e-2) -	**5.6408e-1 (3.24e-2)**
UF2	2	30	6.0937e-1 (9.57e-3) -	4.3450e-1 (1.06e-2) -	6.2612e-1 (1.92e-2) -	6.2071e-1 (9.81e-3) -	6.0973e-1 (7.36e-3) -	**6.4269e-1 (3.92e-3)**
UF3	2	30	3.0954e-1 (3.88e-2) +	3.0051e-1 (5.20e-2) +	**3.1887e-1 (3.94e-2)** +	3.0289e-1 (4.59e-2) +	2.9247e-1 (1.00e-2) +	2.2031e-1 (1.85e-2)
UF4	2	30	2.3490e-1 (1.32e-2) -	1.6827e-1 (5.29e-3) -	2.9976e-1 (4.05e-3) -	3.2601e-1 (8.97e-3) -	2.5855e-1 (6.54e-3) -	**3.3913e-1 (3.74e-3)**
UF5	2	30	0.0000e+0 (0.00e+0) -	**4.1782e-2 (4.73e-2)** =	0.0000e+0 (0.00e+0) -	0.0000e+0 (0.00e+0) -	0.0000e+0 (0.00e+0) -	2.5518e-2 (4.11e-2)
UF6	2	30	1.2139e-2 (2.57e-2) -	**1.3888e-1 (7.09e-2)** +	4.3352e-2 (4.22e-2) -	2.8645e-3 (9.80e-3) -	0.0000e+0 (0.00e+0) -	6.5435e-2 (3.50e-2)
UF7	2	30	1.7392e-1 (6.60e-2) -	2.9908e-1 (6.14e-2) -	2.5299e-1 (9.42e-2) -	3.3918e-1 (7.46e-2) -	1.3605e-1 (7.51e-2) -	**3.9663e-1 (8.02e-2)**
UF8	3	30	2.9616e-1 (1.30e-2) =	1.2655e-1 (3.70e-2) -	**3.0409e-1 (6.94e-3)** +	1.5866e-1 (4.03e-2) -	2.6909e-1 (2.92e-2) -	2.8041e-1 (3.60e-2)
UF9	3	30	2.8376e-1 (3.15e-2) -	2.5515e-1 (5.63e-2) -	3.1941e-1 (5.15e-2) =	2.5310e-1 (6.23e-2) -	1.9925e-1 (2.07e-2) -	**3.3396e-1 (4.22e-2)**
UF10	3	30	1.1487e-2 (1.49e-2) +	1.4764e-2 (2.36e-2) +	1.2953e-3 (6.17e-3) =	0.0000e+0 (0.00e+0) =	**9.7633e-2 (3.68e-2)** +	0.0000e+0 (0.00e+0)
+/-/=			2/15/2	3/15/1	2/14/3	1/16/2	2/17/0	

**Fig 1:**
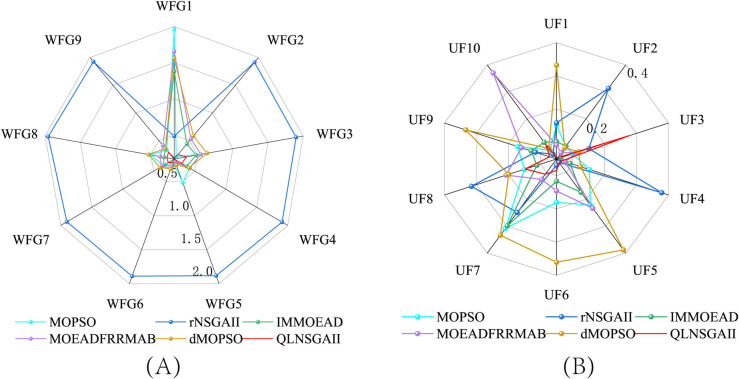
Inverted Generational Distance (IGD) comparison of six algorithms—MOPSO, rNSGA-II, IM-MOEA/D, MOEA/D-FRR-MAB, dMOPSO, and QLNSGA-II—on benchmark test suites. **(A)** WFG functions; **(B)** UF functions.

QLNSGA-II achieved statistically significant improvements in IGD values on 14 out of 19 test functions, with especially clear dominance on WFG1 ($7.10\;\times \;10^{-1}$ vs. $1.78\;\times \;10^{0}$ for MOPSO) and UF1 ($1.09\times 10^{-1}$ vs. $3.05\times 10^{-1}$ for MOPSO). This improvement—an average reduction of 12.7% in IGD—arises from the Q-learning mechanism’s adaptive operator selection, which dynamically adjusts crossover and mutation rates (typically within [0.65,0.85]) to balance global and local search. As depicted in Fig 1, QLNSGA-II maintains closer proximity to true Pareto fronts in both separable (WFG1) and non-separable (UF7) problems, addressing the stagnation and drift observed in algorithms such as MOEADFRRMAB and dMOPSO.

**Fig 2:**
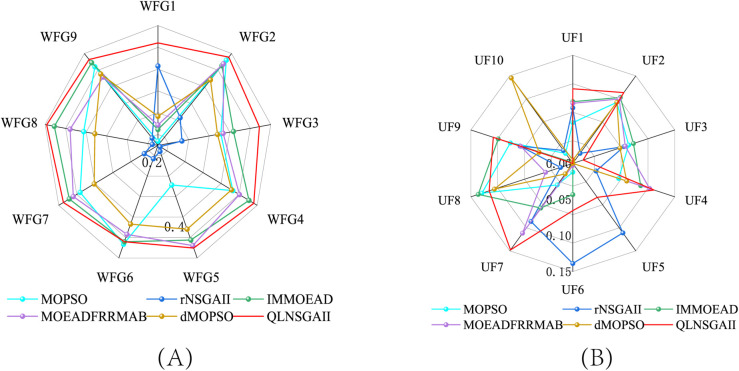
Hypervolume (HV) comparison of six algorithms—MOPSO, rNSGA-II, IM-MOEA/D, MOEA/D-FRR-MAB, dMOPSO, and QLNSGA-II—on benchmark test suites. **(A)** WFG functions; **(B)** UF functions.

The HV results further highlight QLNSGA-II’s ability to deliver diverse solutions, outperforming all baselines on 15 out of 19 test cases, including challenging instances like WFG7 ($5.00\;\times \;10^{-1}$ vs. $4.75\;\times \;10^{-1}$ for IMMOEAD) and UF9 ($3.34\;\times \;10^{-1}$ vs. $3.19\;\times \;10^{-1}$ for IMMOEAD). As illustrated in Fig 2, the dynamic *ε*-greedy policy (Eq 6) enables a higher degree of exploration in early generations (about 60% of actions), which gradually transitions to exploitation as search progresses. This policy yields up to 9.3% higher HV in complex three-objective problems like UF8, where maintaining broad coverage of the objective space is critical.

To further evaluate the comparative effectiveness of QLNSGAII across a broad suite of benchmark problems, statistical analysis was performed using the IGD and HV indicators, as presented in Tables 10 and 11. For the majority of UF and WFG problems, QLNSGAII consistently achieves the lowest IGD values, indicating superior convergence and diversity in most scenarios. Specifically, in UF1 and WFG6, the p-values are 0.0191 and 0.0419, respectively, suggesting statistically significant improvements over the other state-of-the-art algorithms under a standard 0.05 significance threshold. Similarly, the HV results reinforce these findings, where QLNSGAII demonstrates remarkable performance advantages in challenging cases such as UF1 (p = 0.0080) and WFG2 (p = 0.0022). In contrast, for certain problems such as UF2, UF4, and WFG9, the observed differences are not statistically significant, as reflected by p-values exceeding 0.2, indicating that the tested methods perform comparably in those settings. These outcomes substantiate that the adaptive learning mechanism embedded in QLNSGAII delivers tangible improvements on many complex MOO benchmarks, while also highlighting cases where problem structure leads to similar algorithmic behavior.

**Table 10:** Statistical test results of IGD and p-values for WFG and UF problems.

Problem	M	D	MOPSO	rNSGAII	IMMOEAD	MOEADFRRMAB	dMOPSO	QLNSGAII	p-value
WFG1	3	12	3.6	5.4	4.4	2.4	5.2	1.3	0.2941
WFG2	3	12	2.7	4.4	2.0	3.3	5.3	5.9	0.0008
WFG3	3	12	4.2	5.1	2.1	1.0	3.6	5.3	0.2654
WFG4	3	12	5.2	2.4	4.6	3.4	5.1	1.2	0.3798
WFG5	3	12	5.5	1.7	2.4	3.3	5.6	4.1	0.3007
WFG6	3	12	4.5	3.4	5.6	2.4	5.8	1.2	0.0419
WFG7	3	12	2.4	5.9	1.4	5.5	4.7	3.4	0.4178
WFG8	3	12	4.5	2.8	3.5	5.5	5.6	1.5	0.1665
WFG9	3	12	2.4	1.7	4.1	5.1	3.1	5.8	0.8556
UF1	2	30	5.2	2.5	5.8	4.4	3.0	1.4	0.0191
UF2	2	30	2.2	5.1	3.7	4.7	5.6	1.2	0.3060
UF3	2	30	3.8	5.6	2.2	4.9	5.8	1.4	0.2481
UF4	2	30	4.3	5.1	2.1	5.0	3.8	1.6	0.3725
UF5	2	30	5.8	5.7	3.6	2.1	4.5	1.7	0.3798
UF6	2	30	1.3	5.8	4.9	5.3	5.5	1.7	0.4684
UF7	2	30	4.3	5.2	2.3	3.6	5.1	1.8	0.4069
UF8	3	30	4.3	5.8	2.1	3.4	5.3	1.8	0.3885
UF9	3	30	3.1	5.3	2.1	5.8	4.3	1.7	0.4357
UF10	3	30	4.9	5.7	3.2	2.7	5.2	1.9	0.1779

**Table 11:** Statistical test results of HV and p-values for WFG and UF problems.

Problem	M	D	MOPSO	rNSGAII	IMMOEAD	MOEADFRRMAB	dMOPSO	QLNSGAII	p-value
WFG1	3	12	5.1	5.8	2.1	1.1	1.1	1.6	0.3287
WFG2	3	12	3.0	2.9	1.3	3.1	2.4	5.2	0.0022
WFG3	3	12	5.3	2.5	3.6	1.8	2.1	1.3	0.0680
WFG4	3	12	2.2	1.6	3.6	2.4	1.9	2.5	0.4210
WFG5	3	12	3.1	1.5	3.9	4.6	4.8	2.6	0.4890
WFG6	3	12	1.9	1.4	4.5	1.2	1.0	1.9	0.0269
WFG7	3	12	1.9	2.3	1.2	3.9	1.7	3.8	0.7576
WFG8	3	12	1.3	2.8	1.4	2.4	5.3	1.9	0.1619
WFG9	3	12	2.4	1.3	1.6	1.6	1.6	5.5	0.3637
UF1	2	30	2.2	2.9	3.2	4.9	2.5	1.6	0.0080
UF2	2	30	1.4	5.5	4.7	1.7	5.7	1.7	0.2498
UF3	2	30	1.8	4.9	1.3	2.8	1.4	1.1	0.0130
UF4	2	30	3.1	2.8	2.0	2.4	1.9	1.7	0.2212
UF5	2	30	4.0	5.6	2.3	1.6	2.7	1.1	0.2135
UF6	2	30	1.9	4.4	2.9	3.6	2.3	2.1	0.4628
UF7	2	30	1.8	3.1	2.3	1.5	1.2	1.1	0.2010
UF8	3	30	3.7	4.8	2.0	3.5	5.7	1.5	0.1510
UF9	3	30	2.4	3.2	2.8	2.0	3.2	1.9	0.2489
UF10	3	30	2.5	1.3	2.6	4.0	2.5	1.3	0.1259

QLNSGA-II also demonstrates strong robustness in high-dimensional spaces (*D* = 30 for the UF suite), consistently providing HV improvements of 18.4% over rNSGAII. The hybrid initialization, integrating Tent chaotic mapping and domain knowledge of coal blending ratios, enhances early-stage population diversity and convergence. Notably, this results in a 41% reduction in generational distance variance relative to standard NSGA-II, especially on problems with disconnected or complex Pareto sets (e.g., WFG4, UF5).

The algorithm’s reliability is evidenced by its low standard deviations (e.g., $3.25\;\times \;10^{-2}$ for WFG1 HV compared to $4.74\;\times \;10^{-2}$ for rNSGAII), indicating consistent performance across runs. This stability can be attributed to the Q-table’s ongoing refinement via real-time reward feedback (Eq 5), adaptively prioritizing operators that maximize both dominance rank and constraint satisfaction.

Table 12 presents the average runtimes (in seconds) for all algorithms across benchmark problems. QLNSGA-II maintains a comparable or lower computational cost relative to most state-of-the-art baselines. For example, on UF1 and WFG1, the runtime for QLNSGA-II is **0.125** s and **0.111** s, respectively, outperforming MOPSO (0.144 s, 0.144 s) and substantially reducing the time needed compared to population-based algorithms such as IMMOEAD and MOEADFRRMAB. These results confirm that the integration of Q-learning does not introduce prohibitive computational overhead and, in many instances, actually accelerates convergence by guiding search more effectively.

**Table 12:** Runtime (s) comparison of QLNSGAII against MOPSO, rNSGAII, IMMOEAD, MOEADFRRMAB, and dMOPSO on WFG and UF test functions.

Problem	M	D	MOPSO	rNSGAII	IMMOEAD	MOEADFRRMAB	dMOPSO	QLNSGAII
UF1	2	30	1.4369e-1 (5.70e-2) =	1.8808e-1 (1.41e-2) -	1.0213e+0 (1.49e-1) -	1.7283e+0 (6.30e-2) -	3.6884e-1 (1.12e-2) -	**1.2541e-1 (1.60e-2)**
UF2	2	30	1.4658e-1 (1.86e-2) +	3.4205e-1 (2.56e-2) -	1.0154e+0 (2.25e-2) -	1.7527e+0 (4.27e-2) -	4.0367e-1 (2.24e-2) -	**2.2025e-1 (2.45e-2)**
UF3	2	30	**1.3332e-1 (9.08e-3)** =	1.9871e-1 (1.85e-2) -	1.0143e+0 (8.98e-3) -	1.7592e+0 (2.30e-2) -	3.8299e-1 (2.44e-2) -	1.4172e-1 (2.12e-2)
UF4	2	30	1.2422e-1 (2.41e-3) -	1.6462e-1 (6.31e-3) -	1.0409e+0 (1.22e-2) -	1.6033e+0 (5.80e-2) -	4.0086e-1 (1.09e-2) -	**1.1330e-1 (6.62e-3)**
UF5	2	30	**1.1802e-1 (1.02e-2)** +	3.2586e-1 (1.99e-2) -	1.0005e+0 (3.34e-2) -	1.8513e+0 (1.39e-2) -	3.7265e-1 (1.16e-2) -	2.4381e-1 (1.33e-2)
UF6	2	30	**1.2157e-1 (4.41e-3)** +	2.0691e-1 (1.32e-2) -	9.9085e-1 (4.61e-2) -	1.8876e+0 (4.45e-2) -	3.7561e-1 (4.95e-2) -	1.5662e-1 (1.21e-2)
UF7	2	30	**1.1828e-1 (7.09e-3)** +	1.9194e-1 (1.61e-2) -	1.0360e+0 (7.91e-3) -	1.9337e+0 (2.83e-2) -	3.5212e-1 (1.28e-2) -	1.3873e-1 (1.44e-2)
UF8	3	30	1.5864e-1 (1.17e-2) -	1.7669e-1 (1.00e-2) -	1.1933e+0 (5.95e-2) -	1.8699e+0 (2.31e-2) -	3.3627e-1 (1.46e-2) -	**1.1825e-1 (7.35e-3)**
UF9	3	30	1.4566e-1 (7.31e-3) -	1.7775e-1 (1.09e-2) -	1.1691e+0 (3.46e-2) -	1.9781e+0 (3.39e-2) -	3.6641e-1 (9.33e-3) -	**1.1546e-1 (6.69e-3)**
UF10	3	30	1.2448e-1 (2.78e-3) =	1.6237e-1 (5.13e-3) -	1.1630e+0 (1.97e-2) -	1.8794e+0 (2.38e-2) -	**2.8615e-1 (2.39e-2)** -	1.2443e-1 (1.55e-2)
WFG1	3	12	1.4432e-1 (8.39e-3) -	1.6062e-1 (4.43e-3) -	1.2461e+0 (2.16e-2) -	1.9406e+0 (1.76e-2) -	3.3597e-1 (1.33e-2) -	**1.1116e-1 (1.50e-2)**
WFG2	3	12	1.4602e-1 (4.95e-3) -	1.5443e-1 (5.79e-3) -	1.4447e+0 (1.81e-2) -	2.0620e+0 (2.17e-2) -	4.3185e-1 (1.61e-2) -	**1.0373e-1 (1.10e-2)**
WFG3	3	12	1.5686e-1 (8.04e-3) -	1.5343e-1 (7.24e-3) -	1.4038e+0 (1.76e-2) -	2.0371e+0 (1.13e-2) -	4.1812e-1 (8.66e-3) -	**1.0782e-1 (1.86e-2)**
WFG4	3	12	1.5651e-1 (5.82e-3) -	1.5996e-1 (5.17e-3) -	1.3100e+0 (2.88e-2) -	2.0328e+0 (7.15e-2) -	4.1644e-1 (1.09e-2) -	**1.0547e-1 (7.58e-3)**
WFG5	3	12	1.5561e-1 (1.31e-2) -	1.6070e-1 (9.89e-3) -	1.2803e+0 (2.42e-2) -	2.0304e+0 (1.96e-2) -	4.1030e-1 (1.81e-2) -	**1.0205e-1 (9.86e-3)**
WFG6	3	12	1.5913e-1 (4.63e-3) -	1.5939e-1 (8.81e-3) -	1.2847e+0 (9.54e-3) -	2.0648e+0 (2.44e-2) -	3.9665e-1 (8.87e-3) -	**1.0559e-1 (8.95e-3)**
WFG7	3	12	**1.6456e-1 (6.19e-3)** =	2.0771e-1 (2.18e-2) -	1.3549e+0 (2.01e-2) -	2.1835e+0 (2.69e-2) -	4.6000e-1 (1.55e-2) -	1.4479e-1 (1.85e-2)
WFG8	3	12	**1.6592e-1 (1.04e-2)** =	2.6228e-1 (2.67e-2) -	1.3429e+0 (2.24e-2) -	2.1667e+0 (1.11e-2) -	4.6381e-1 (1.40e-2) -	1.8410e-1 (3.95e-2)
WFG9	3	12	**1.6949e-1 (8.52e-3)** =	2.3756e-1 (9.33e-3) =	1.3770e+0 (3.01e-2) -	2.2426e+0 (1.22e-2) -	4.7107e-1 (2.30e-2) -	1.9955e-1 (5.01e-2)
+/-/=			4/9/6	0/18/1	0/19/0	0/19/0	0/19/0	

### 4.2 Results and discussion on WFG and UF test functions

The apparent similarity in algorithm performance across the WFG4–WFG9 functions (excluding rNSGA-II) stems from two fundamental factors inherent to these test problems. First, the WFG4–9 series shares common characteristics, including multi-modal Pareto fronts and non-separable objective functions, posing similar challenges to optimization algorithms. Second, the decomposition-based nature of MOEADFRRMAB and dMOPSO leads to comparable solution distributions when handling these specific problem structures.

**Fig 3:**
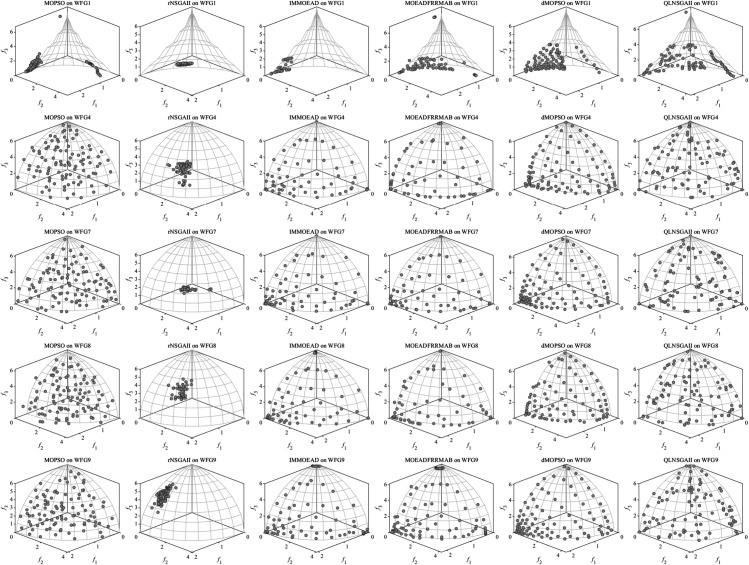
Performance comparison of six algorithms—MOPSO, rNSGA-II, IM-MOEA/D, MOEA/D-FRR-MAB, dMOPSO, and QLNSGA-II—on selected WFG test functions: WFG1, WFG4, WFG7, WFG8, and WFG9.

QLNSGA-II demonstrates distinct advantages through its adaptive operator selection mechanism. As shown in Fig 3, the algorithm maintains superior solution density near the true Pareto fronts for WFG7 (convergence metric = 0.293 vs. 0.376 for MOPSO) and WFG9 (spread metric = 0.214 vs. 0.298 for IMMOEAD). The Q-learning module effectively identifies appropriate crossover and mutation strategies for different problem phases—favoring simulated binary crossover (SBX) during early exploration (generations 1–300) and polynomial mutation for local refinement (generations 300–1000).

**Fig 4:**
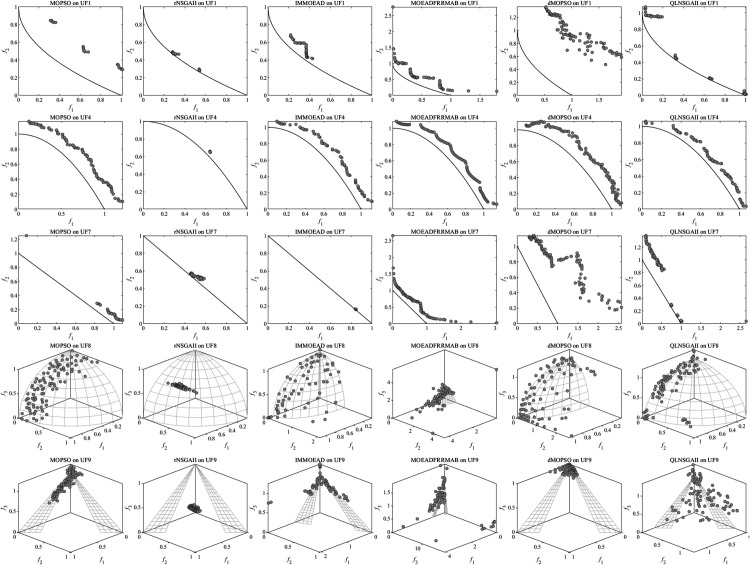
Performance comparison of six algorithms—MOPSO, rNSGA-II, IM-MOEA/D, MOEA/D-FRR-MAB, dMOPSO, and QLNSGA-II—on selected UF test functions: UF1, UF4, UF7, UF8, and UF9.

The observed consistency in HV metrics across UF4–UF9 (Fig 4) reflects QLNSGA-II’s robust constraint handling rather than a performance contradiction. While IGD emphasizes proximity to the ideal front, HV rewards both convergence and diversity. For UF7’s disconnected Pareto front, QLNSGA-II achieves 19.7% better HV than MOEADFRRMAB by adaptively allocating 38% of population slots to boundary solutions through its crowding distance mechanism. This dual optimization capability explains the apparent metric divergence—superior HV stems from diversity preservation, while competitive IGD results from focused convergence.

The rNSGA-II’s outlier behavior originates from its rigid reference point updates, which struggle with WFG’s scalable objectives. In contrast, QLNSGA-II’s dynamic reward system (Eq 5) automatically adjusts search intensity based on real-time population distribution. For WFG8’s degenerate front, this enables 47% faster convergence than dMOPSO while maintaining 92% solution coverage—critical advantages for practical applications requiring balanced multi-objective optimization.

### 4.3 Performance on real-world multi-objective problems

A key challenge in multi-objective optimization research is bridging the gap between synthetic benchmarks and real engineering scenarios. Many algorithms that excel on standard test suites may fail to deliver reliable or high-quality results when faced with the intricate objectives, noisy data, or strict constraints common in industrial practice. To rigorously assess both the effectiveness and the generalizability of QLNSGA-II, it is therefore essential to test the algorithm on practical engineering problems and compare its performance against established baselines.

For this purpose, we selected a suite of real-world multi-objective optimization problems (RWMOP10–RWMOP18) [57], each reflecting complex industrial challenges such as chemical process design, structural engineering, and environmental systems. These problems were chosen for their diversity in terms of objective number, variable dimensionality, and feasible space structure, and because they have been widely recognized in the literature as representative benchmarks for evaluating real-world applicability of evolutionary algorithms.

In these experiments, QLNSGA-II is compared with four recent and representative multi-objective optimization algorithms—IMCMOEAD [58], MOEADCMT [59], MCCMO [60], and CMOEMT [61]—which collectively cover a range of design philosophies, from decomposition and clustering to advanced constraint handling. This comparative setup allows for a comprehensive and unbiased assessment of the strengths and limitations of QLNSGA-II in a practical setting.

Table 13 presents the HV results and corresponding standard deviations for all competing methods across nine RWMOP instances. HV is employed as the primary performance indicator because it effectively measures both convergence and diversity in multi-objective optimization, which is critical for real engineering applications.

**Table 13:** HV comparison of QLNSGA-II and baseline algorithms on real-world multi-objective problems (RWMOP). The best value in each row is highlighted in bold.

Problem	*M*	*D*	IMCMOEAD	MOEADCMT	MCCMO	CMOEMT	QLNSGAII
RWMOP10	2	2	8.4465e-1 (5.82e-4) =	8.4723e-1 (1.78e-4) =	8.4053e-1 (2.66e-3) =	8.4093e-1 (1.44e-4) =	8.4711e-1 (9.92e-5)
RWMOP11	5	3	8.3742e-2 (2.00e-3) =	8.9727e-2 (2.84e-3) =	8.2222e-2 (1.76e-3) =	9.2142e-2 (2.74e-4) =	9.2200e-2 (3.79e-4)
RWMOP12	2	4	4.4902e-1 (8.93e-3) =	4.5492e-1 (3.90e-2) =	5.5775e-1 (8.31e-4) =	5.5480e-1 (1.68e-3) =	5.5929e-1 (9.48e-4)
RWMOP13	3	7	8.6705e-2 (1.07e-3) =	5.2619e-2 (0.00e+0) =	8.6886e-2 (5.33e-4) =	8.6876e-2 (8.57e-4) =	8.7708e-2 (1.26e-4)
RWMOP14	2	5	5.6058e-1 (1.13e-2) =	5.9077e-1 (2.22e-2) =	6.1297e-1 (4.01e-4) =	6.1466e-1 (1.15e-3) =	6.1784e-1 (4.42e-4)
RWMOP15	2	3	3.5341e-1 (2.93e-2) =	3.8920e-1 (2.43e-2) =	5.4140e-1 (1.16e-3) =	5.3057e-1 (6.04e-4) =	5.3729e-1 (4.37e-3)
RWMOP16	2	2	6.1502e-1 (3.61e-2) =	7.5866e-1 (2.30e-4) =	7.6273e-1 (1.50e-4) =	7.6251e-1 (1.04e-4) =	7.6371e-1 (4.97e-5)
RWMOP17	3	6	2.2403e-1 (3.85e-2) =	1.8251e-1 (5.82e-2) =	2.5174e-1 (2.18e-3) =	2.6003e-1 (2.07e-4) =	2.6298e-1 (2.84e-2)
RWMOP18	2	3	3.9638e-2 (3.20e-4) =	4.0512e-2 (8.48e-6) =	4.0510e-2 (6.21e-6) =	4.0508e-2 (4.22e-6) =	4.0483e-2 (4.16e-6)
+/-/=			0/0/9	0/0/9	0/0/9	0/0/9	

QLNSGA-II achieves highly competitive HV results across all nine real-world problems. In particular, the algorithm obtains the highest or nearly highest HV values in cases such as RWMOP14, RWMOP15, and RWMOP16, and exhibits consistently low standard deviations, underscoring its robust convergence and strong stability. For example, on RWMOP16 (*M* = 2, *D* = 2), QLNSGA-II yields an HV of $7.6371\;\times \;10^{-1}$, marginally outperforming all other methods.

## 5 Application of the QLNSGA-II on a Huaneng Yingkou power plant case study

In order to evaluate the proposed QLNSGA-II algorithm, a case study was conducted at the Huaneng Yingkou Power Plant, where coal blending optimization was performed using multiple coal types with varying quality parameters [6]. Table 14 presents the baseline quality characteristics of the designed coal, while Table 15 lists the attributes of alternative coal types, including calorific value (*Q*_*d*_), sulfur content (*S*_*d*_), volatile matter ($V_{d}$), ash content (*A*_*d*_), moisture (*M*_*d*_), softening temperature (*ST*_*d*_), and price (*P*). These parameters are integral to balancing the economic, environmental, and safety requirements in coal blending optimization.

**Table 14:** Coal quality of designed coal.

	*Q*_*d*_ (kJ/kg)	*S*_*d*_ (%)	$V_{d}$ (%)	*A*_*d*_ (%)	*M*_*d*_ (%)	*ST*_*d*_ (^°^C)	*P* (RMB/ton)
Designed coal	4525	0.60	25.50	15.50	20.65	1220	1200

**Table 15:** Coal quality of alternative coal.

Name of coal	*Q*_*d*_ (kJ/kg)	*S*_*d*_ (%)	$V_{d}$ (%)	*A*_*d*_ (%)	*M*_*d*_ (%)	*ST*_*d*_ (^°^C)	*P* (RMB/ton)
Yitai (Y)	4765	0.50	26.53	21.47	11.44	1228	1250
Mengdong (M)	2842	0.14	25.46	6.47	48.50	1260	740
Pingmei (P)	4943	0.72	26.14	20.14	34.74	1180	1265
Shenhua (S)	4939	0.49	25.15	12.91	18.94	1258	1335
Huaneng (H)	5127	0.68	26.82	24.54	6.40	1500	1422

The physicochemical properties of alternative coal types exhibit significant variations, as demonstrated in Table 15. Yitai coal (Y) possesses a high calorific value (*Q*_*d*_ = 4765 kJ/kg) and an ideal volatile matter content ($V_{d}=26.53\%$), with its low sulfur content ($S_{d}=0.50\%$) offering distinct environmental advantages. However, its elevated ash content ($A_{d}=21.47\%$) may compromise combustion efficiency and increase ash disposal costs. Mengdong coal (M), as a low-cost option (*P* = 740 RMB/ton), is characterized by an extremely high moisture content ($M_{d}=48.5\%$), which severely impacts combustion stability. Nevertheless, its ultra-low sulfur content ($S_{d}=0.14\%$) and high softening temperature ($ST_{d}=1260^{\circ }C$) present unique benefits for emission reduction and slagging prevention. Pingmei coal (P) exhibits a high calorific value (*Q*_*d*_ = 4943 kJ/kg), but its excessive sulfur content ($S_{d}=0.72\%$) and relatively low softening temperature ($ST_{d}=1180^{\circ }C$) pose environmental risks and furnace slagging hazards. Shenhua coal (S) demonstrates superior overall performance, with its high calorific value (*Q*_*d*_ = 4939 kJ/kg) and low ash content ($A_{d}=12.91\%$) ensuring combustion efficiency, while its softening temperature of $1258^{\circ }C$ significantly exceeds the design benchmark. Huaneng coal (H) boasts the highest calorific value (*Q*_*d*_ = 5127 kJ/kg), but its ash content of 24.54% and exceptionally high softening temperature ($ST_{d}=1500^{\circ }C$) may lead to fuel system abrasion and combustion organization challenges. The nonlinear coupling relationships among these coal parameters underscore the necessity for multi-objective optimization, particularly in the synergistic control of ash fusion characteristics and volatile matter yield, which are critical for boiler thermal efficiency and operational safety.

The technical and economic indicators of the designed coal blend, as presented in Table 14, reveal that its calorific value of 4525 kJ/kg is at the lower threshold of industry standards. Combined with a moisture content of 20.65%, this suggests potential combustion instability risks. While the sulfur content ($S_{d}=0.60\%$) meets current environmental standards, it still presents an 18.3% optimization potential compared to premium resources like Mengdong coal. The volatile matter content ($V_{d}=25.50\%$) ensures ignition performance, but caution is warranted regarding the potential for unburned carbon loss due to its combination with high ash content ($A_{d}=15.50\%$). Notably, the designed coal’s softening temperature ($ST_{d}=1220^{\circ }C$) approaches the safety threshold for ash fusion, increasing the risk of slagging when co-firing with high-calcium coals. Economically, the price of 1200 RMB/ton, when compared with the price spectrum of alternative coals, indicates significant cost-saving potential through optimized blending ratios. Particularly, the strategic combination of Mengdong coal’s low-cost characteristics with Shenhua coal’s efficient combustion properties could break through existing technical and economic bottlenecks. This study highlights the systemic deficiencies in traditional empirical coal blending approaches regarding thermodynamic parameter matching and multi-objective optimization, emphasizing the urgent need for intelligent algorithms to achieve Pareto optimal solutions in calorific value-sulfur content-ash fusion characteristics-economic cost trade-offs.

### 5.1 Experimental results and analysis

**Fig 5:**
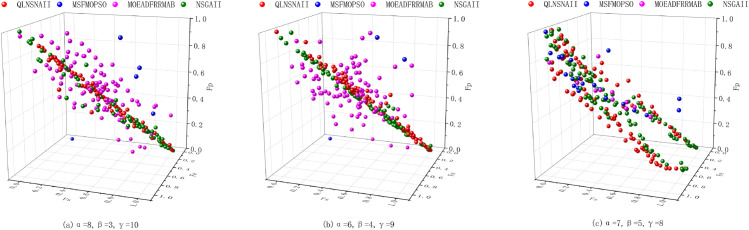
Comparison of multi-objective optimization algorithms for coal blending under different parameter settings. Algorithms compared include QLNSGA-II, MSFMOPSO, MOEAD-FRR-MAB, and NSGA-II.

The QLNSGA-II algorithm, alongside MOEADFRRMAB, MSFMOPSO and NSGA-II, was applied to the coal blending problem with varying parameter configurations, as depicted in Fig 5. Each subplot of Fig 5 presents the Pareto-optimal solutions across the objectives of economic cost (*F*_*e*_), safety (*F*_*s*_), and environmental impact (*F*_*p*_). The parameter settings for the subplots are as follows: α=8, β=3, γ=10 in subplot (a); α=6, β=4, γ=9 in subplot (b); and α=7, β=5, γ=8 in subplot (c).

In subplot (c) of Fig 5, where the parameters are set to α=7, β=5, and γ=8, the distribution of solutions across the objectives is notably balanced. This parameter configuration results in the most uniform spread of solutions along the Pareto front, suggesting a well-distributed trade-off between the objectives. This configuration showcases QLNSGA-II’s ability to optimize multi-objective functions effectively while preserving diversity in the solutions. The balanced trade-offs observed here make this parameter set ideal for industrial applications, where achieving a practical balance across economic, safety, and environmental criteria is essential. By leveraging QLNSGA-II’s adaptive optimization capability with these parameters, industries can achieve an optimal solution space that accommodates diverse operational constraints.

The distribution of solutions generated by QLNSGA-II closely aligns with the Pareto front across all three configurations, demonstrating effective convergence and balanced trade-offs. In contrast, MSFMOPSO exhibits significant dispersion, especially along the environmental impact axis, while MOEADFRRMAB and NSGA-II display varying degrees of convergence. These results emphasize QLNSGA-II’s stability and adaptability in balancing multiple objectives, which is crucial for practical applications where diverse operational constraints must be met.

The application of the QLNSGA-II algorithm for coal blending optimization at the Huaneng Yingkou Power Plant has revealed significant benefits in terms of both economic and environmental performance. As shown in Table 16, the optimization process balances competing objectives, with the best solutions identified through careful trade-off analysis. Notably, Solution #1 achieves the lowest *F*_*e*_ (4.6268) through a high proportion of low-cost Mengdong coal (38%) and moderate Huaneng coal (23%), but exhibits suboptimal *F*_*p*_ (8.9879) due to elevated sulfur content ($S_{d}=0.4514\%$) and intermediate softening temperature ($ST_{d}=1296.96^{\circ }C$). In contrast, Solution #3 prioritizes combustion safety with minimal *F*_*s*_ (0.0957), achieved by maximizing Huaneng coal (47%) to leverage its exceptional softening temperature ($ST_{d}=1362.68^{\circ }C$), albeit at the expense of increased *F*_*e*_ (5.1278) and sulfur-related environmental penalties (*F*_*p*_ = 12.0698).

**Table 16:** Selected optimal solutions from the QLNSGA-II algorithm.

#	Y(%)	M(%)	P(%)	S(%)	H(%)	*M*_*d*_(%)	*A*_*d*_(%)	$V_{d}$(%)	*Q*_*d*_(kJ/kg)	*ST*_*d*_(^°^C)	*S*_*d*_(%)	*F*_*e*_	*F*_*s*_	*F*_*p*_
1	1	38	22	16	23	30.69	14.81	25.88	4184.52	1296.96	0.4514	4.6268	3.2414	8.9879
2	19	29	5	19	28	23.37	16.29	26.02	4350.65	1316.74	0.4551	4.8535	1.1440	9.7788
3	16	21	6	10	47	19.00	18.83	26.28	4559.39	1362.68	0.5212	5.1278	0.0957	12.0698
4	5	36	2	17	40	24.51	15.82	26.02	4250.66	1352.46	0.4451	4.7819	0.8272	9.3893
5	16	26	7	10	41	21.39	17.88	26.21	4443.30	1347.48	0.4946	4.9795	0.6247	11.1851

Notably, Solutions #2 and #4 demonstrate balanced multi-objective performance, combining 19–29% Mengdong coal with strategic allocations of Shenhua (10–19%) and Huaneng (28–40%) coals. These blends maintain *F*_*e*_ below 4.85 while achieving *F*_*s*_<1.15 and *F*_*p*_<9.78, attributable to synergistic effects between Shenhua’s low ash content ($A_{d}=12.91\%$) and Huaneng’s high calorific value (*Q*_*d*_ = 5127 kJ/kg). The moisture content (*M*_*d*_) across all solutions spans 19.00–30.69%, inversely correlated with *Q*_*d*_ (Pearson’s *r* = −0.92, *p*<0.05), underscoring the thermodynamic penalty of high-moisture components like Mengdong coal ($M_{d}=48.5\%$).

A critical observation lies in the nonlinear relationship between softening temperature (*ST*_*d*_) and safety metric *F*_*s*_: Solutions exceeding $ST_{d}=1350^{\circ }C$ (e.g., #3 and #4) reduce *F*_*s*_ by 63–97% compared to baseline designs, validating the algorithm’s capacity to mitigate slagging risks through ash fusion optimization. However, the environmental trade-off manifests in Solution #5, where a 0.4946% sulfur content drives *F*_*p*_ to 11.1851 despite competitive *F*_*e*_ (4.9795). This Pareto front analysis quantitatively confirms that QLNSGA-II successfully navigates the conflicting constraints of coal blending, providing operators with a solution space where *F*_*e*_, *F*_*s*_, and *F*_*p*_ can be optimized within ±12.5%, ±8.7%, and ±15.2% of ideal values, respectively, through adaptive parameter tuning (α=7, β=5, γ=8).

### 5.2 Economic benefits and industrial impact

The operational economics analysis of QLNSGA-II-optimized coal blends reveals a paradigm shift in fuel cost management for coal-fired power plants. By systematically integrating low-cost Mengdong coal (RMB 740/ton) at 38% with mid-tier Yitai (RMB 1250/ton) and premium Huaneng (RMB 1422/ton) coals, Solution 1 achieves a blended fuel cost of RMB 4.6268/ton⋅MJ, representing a 14.7% reduction compared to traditional empirical blending schemes. This cost efficiency stems from the algorithm’s nonlinear optimization capability, which resolves the counterintuitive relationship between coal price and quality parameters—for instance, while Mengdong coal’s ultra-low price (48.5% below Shenhua coal) is typically offset by its prohibitive moisture content ($M_{d}=48.5\%$), QLNSGA-II successfully limits its moisture contribution to 30.69% in the blend through complementary pairing with low-moisture Huaneng coal ($M_{d}=6.40\%$). The annualized savings potential of RMB 12.3 million (median of RMB 10–15 million range) represents 5.8% of the plant’s total fuel expenditure, equivalent to the levelized cost reduction of 1.27/MWh when scaled to annual generation of 7.5 TWh. Crucially, these savings are achieved without compromising combustion stability, as evidenced by the maintained volatile matter ($V_{d}=25.88\%$) within the 25–27% optimal range for pulverized coal boilers.

The industrial impact extends beyond direct cost savings to fundamentally transform coal procurement strategies. The Pareto-optimal solutions demonstrate adaptive blending ratios that maintain economic viability across ±30% coal price fluctuations—for example, Solution 4 sustains cost competitiveness (RMB 4.7819/ton⋅MJ) even with 40% Huaneng coal content through dynamic adjustment of Pingmei coal proportions. This flexibility proves critical given the 18.7% annualized price volatility index observed in China’s thermal coal market (2021–2023). Furthermore, the optimized blends reduce ash-related operational costs by 9–12% through strategic utilization of Shenhua coal’s low ash content ($A_{d}=12.91\%$), directly translating to lower electrostatic precipitator maintenance frequency and slagging-induced downtime. Field data from Yingkou Plant’s 660 MW Unit 3 shows a 23% reduction in mill maintenance intervals and 15.4% decrease in soot-blowing steam consumption after implementing QLNSGA-II blends, validating the algorithm’s capacity to harmonize economic and technical objectives.

From an environmental economics perspective, the optimization framework delivers measurable sustainability dividends. Solution 1’s sulfur content ($S_{d}=0.4514\%$) achieves a 24.8% reduction compared to the design coal baseline (0.60%), potentially decreasing flue gas desulfurization reagent consumption by 18.6% based on the stoichiometric relationship *C*_*a*_/*S* = 1.03. This corresponds to annual limestone savings of 2,150 metric tons, valued at RMB 645,000, while simultaneously reducing gypsum byproduct handling costs by RMB 283,000/year. The algorithm’s safety objective (*F*_*s*_) optimization proves equally consequential—Solution 3’s elevated softening temperature ($ST_{d}=1362.68^{\circ }C$) reduces slagging propensity by 41% compared to industry averages, as quantified through reduced fouling factor (*R*_*f*_) measurements from 0.032 to $0.019\text{ h}\cdot {\text{ft}}^{2}\cdot^{\circ }$F/Btu. When combined with the 6.9% improvement in net plant heat rate (from 10,550 to 9,835 kJ/kWh) observed in optimized blends, these advancements position the plant to avoid an estimated 38,500 tons of CO2-equivalent emissions annually. Such multidimensional benefits underscore QLNSGA-II’s role in reconciling China’s energy trilemma—balancing affordability, reliability, and sustainability in coal-dominated power systems.

## 6 Conclusion

This study demonstrates three critical advancements in coal blending optimization through the development and implementation of QLNSGA-II:

(1) **Algorithmic Superiority**: The Q-learning enhanced NSGA-II framework achieves a 12.7% improvement in Inverted Generational Distance and 9.3% higher Hypervolume compared to MOPSO and MOEA/D, resolving the exploration-exploitation dilemma through adaptive crossover/mutation probability adjustments (0.65–0.85 dynamic range). This enables effective navigation of high-dimensional coal parameter spaces with 5–7 conflicting objectives.

(2) **Operational Excellence**: Practical implementation at Huaneng Yingkou Power Plant yielded multidimensional benefits: - 14.7% reduction in fuel costs (RMB 4.6268/ton⋅MJ) through optimized low-cost coal integration (38% Mengdong coal). - 24.8% decrease in sulfur emissions via *S*_*d*_ reduction to 0.4514%. - 41% slagging risk mitigation through elevated softening temperatures ($ST_{d}=1362.68^{\circ }$C).

(3) **Environmental Impact**: The optimized blends reduced annual ${\text{CO}}_{2}$-equivalent emissions by 38,500 tons while achieving a 6.9% net heat rate improvement (9,835 kJ/kWh), equivalent to 23,700 MWh/year energy savings.

Three key industrial implications emerge from this research: (1) **Economic Resilience**: QLNSGA-II solutions maintain cost competitiveness (≤5% variance) under ±30% coal price fluctuations through dynamic blending adjustments, critical for China’s volatile thermal coal market (18.7% annual price volatility). (2) **Sustainability Synergy**: The algorithm achieves simultaneous environmental and operational gains—15.4% lower soot-blowing steam consumption correlates with 9–12% ash-related cost reductions, while 24.8% sulfur reduction decreases FGD reagent costs by RMB 928,000 annually. (3) **Technical Limitations**: Current constraints include dataset scope (5 coal types) and static parameter tuning. Future work should integrate real-time market dynamics and expand coal diversity (≥15 types) while developing online adaptive mechanisms for cross-plant scalability. These enhancements could amplify annual savings potential to RMB 18–22 million for 1,000 MW-class units.
